# Modeling pre-metastatic lymphvascular niche in the mouse ear sponge assay

**DOI:** 10.1038/srep41494

**Published:** 2017-01-27

**Authors:** Melissa García-Caballero, Maureen Van de Velde, Silvia Blacher, Vincent Lambert, Cédric Balsat, Charlotte Erpicum, Tania Durré, Frédéric Kridelka, Agnès Noel

**Affiliations:** 1 Laboratory of Tumor and Developmental Biology, GIGA-Cancer, University of Liège, Sart-Tilman, B-4000, Liège, Belgium; 2 Department of Obstetrics and Gynecology, CHU Liège, Sart-Tilman, B-4000, Liège, Belgium

## Abstract

Lymphangiogenesis, the formation of new lymphatic vessels, occurs in primary tumors and in draining lymph nodes leading to pre-metastatic niche formation. Reliable *in vivo* models are becoming instrumental for investigating alterations occurring in lymph nodes before tumor cell arrival. In this study, we demonstrate that B16F10 melanoma cell encapsulation in a biomaterial, and implantation in the mouse ear, prevents their rapid lymphatic spread observed when cells are directly injected in the ear. Vascular remodeling in lymph nodes was detected two weeks after sponge implantation, while their colonization by tumor cells occurred two weeks later. In this model, a huge lymphangiogenic response was induced in primary tumors and in pre-metastatic and metastatic lymph nodes. In control lymph nodes, lymphatic vessels were confined to the cortex. In contrast, an enlargement and expansion of lymphatic vessels towards paracortical and medullar areas occurred in pre-metastatic lymph nodes. We designed an original computerized-assisted quantification method to examine the lymphatic vessel structure and the spatial distribution. This new reliable and accurate model is suitable for *in vivo* studies of lymphangiogenesis, holds promise for unraveling the mechanisms underlying lymphatic metastases and pre-metastatic niche formation in lymph nodes, and will provide new tools for drug testing.

The lymphatic vascular system plays a key role in the regulation of tissue homeostasis and in the control of interstitial fluid pressure and lipid metabolism. Although normally quiescent in adults^1^, the outgrowth of new lymphatic vessels from pre-existing ones (lymphangiogenesis) occurs in numerous pathologies including cancers, inflammatory diseases, fibrosis, and graft transplant rejection^2^,^3^. Lymphatic vessels are recognized as important regulators of immunity and inflammation^4^, and represent an important route for metastatic dissemination^5^. Tumor-associated lymphangiogenesis has potential significance not only at the primary site but also in the draining lymph nodes (LNs), which still remain the first site of metastasis in several human malignancies, such as cutaneous malignant melanoma and carcinomas. Indeed, lymphangiogenesis within draining LNs contributes to enhanced distant organ metastases^6^ and metastatic LN reflect poor prognosis for patients. Importantly, the drainage of tumor-derived factors through the lymphatic system to regional LNs plays an important role in conditioning LN microenvironment before the arrival of disseminated tumor cells. This cross-talk between the primary tumor and the sentinel LNs induces profound changes in LNs, elaborating a pre-metastatic niche receptive and supportive to metastatic cancer cells^7^. Although pre-metastatic changes in LNs have been documented in experimental models^8^, little is known about the mechanisms underlying lymphangiogenesis, which occurs during LN pre-metastatic niche formation.

A current limitation to progress in lymphangiogenesis research has been the lack of simple, reliable, reproducible, and quantitative assays of the lymphangiogenic response *in vivo* that take into account the complexity of this biological process. The obvious advantage of examining lymphangiogenesis *in vivo* is to recapitulate the sprouting of lymphatic endothelial cells (LEC) from pre-existing vessels in different environments (primary tumor, draining LNs). The classical *in vivo* assays for lymphangiogenesis include at least tumor transplantation^9^, corneal assays^10^,^11^, spheroid-based human microvessel formation^12^, and genetic models of lymphatic vessel development (zebrafish, tadpole)^13^,^14^. The matrigel plug assay initially developed more than 20 years ago to assess angiogenesis^15^, is now also used to study lymphangiogenesis^16^,^17^. However, the main drawback of these *in vivo* models is the difficult quantification and/or the high intra-experimental variability. Furthermore, matrigel is not a native extracellular environment for LEC, which are surrounded by discontinuous basal membrane and directly in contact with the interstitial matrix^2^. There is an urgent need for new accurate *in vivo* models to delineate the cross-talk established between malignant cells and their microenvironment in the primary tumor and in draining LNs at a pre-metastatic stage. A more comprehensive understanding of molecular signals involved in the elaboration of a pre-metastatic LN niche that facilitates tumor metastasis is mandatory to identify biomarkers and/or provide novel therapeutic strategies aimed at preventing LN metastasis. This is essential not only to decrease the risk of systemic tumor spread in patients with lymphatic metastasis, but also to improve the effectiveness of new treatments.

In this paper, we designed a gelatin sponge-based assay optimized for close reconstitution of sprouting lymphangiogenesis in the primary tumor, and of lymphatic vasculature remodeling in pre-metastatic and metastatic LNs. The mouse ear sponge assay takes advantage of the rich lymphatic vasculature presents in adult mouse ears. In this system, a gelatin sponge soaked with tumor cells and embedded in a collagen matrix, is implanted between the two mouse ear skin layers. In this work, we use the B16F10 melanoma cell line because of its great ability to invade the lymphatic system. We demonstrate that B16F10 melanoma cells confined within the biomaterial do not directly spread through lymphatic vessels after their implantation. The bioluminescent evaluation of reporter gene activity in engineered tumor cells allows to track metastatic tumor cells and to analyze lymphangiogenesis in LNs prior (pre-metastatic stage) or after (metastatic stage) metastatic dissemination. We provide a robust computerized-assisted quantification method to characterize in-depth the initial lymphatic vasculature in the primary tumor microenvironment (ears) and in LNs at different stages. In addition to providing a novel reliable model, our data give evidence for an important remodeling of the lymphatic vasculature in pre-metastatic LNs, which is characterized by the reorganization of the vessels’ spatial distribution.

## Results

### Mouse ear gelatin sponges *versus* intradermal injections

**Figure 1:**
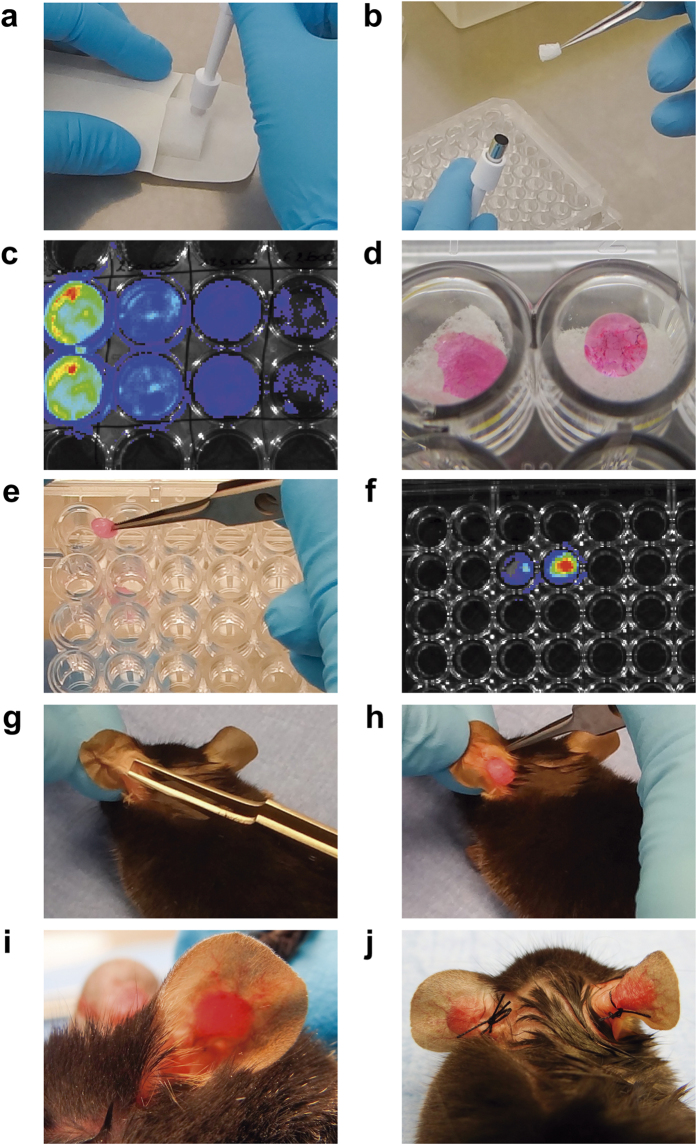
Description of the main steps of the ear sponge assay. The two major steps of the ear sponge assay are the sponge preparation (**a**–**f**) and the sponge implantation in mouse ears (**g**–**j**). (**a**) Sterile compressed gelatin sponges were cut with a sterile biopsy punch into small cylindrical pieces. (**b**) Sponges were next placed in a 96-well plate (one per well) with a forceps. (**c**) For each experiment, the positivity for the *Luciferase* gene expression in B16F10Luc+ transfected cells was checked by bioluminescence. Serial dilutions of cells, starting at 5 × 10^5^ B16F10Luc+ cells (wells at left), were associated with proportional Xenogen signals. (**d**) A drop of the appropriate cell suspension (20 μl) was seeded on top of the sponge, allowing the progressive diffusion of the solution into the sponge during an incubation for 30 minutes at 37 °C. (**e**) Sponges were soaked with a collagen mix, placed immediately in a new well and incubated at 37 °C for 30 minutes to allow collagen gel polymerization. (**f**) Such collagen coating did not affect the bioluminescence emitted by cells when luciferin was added inside the sponge. In the picture, sponges soaked with 20 μl of 1 × 10^5^ and 2 × 10^5^ at left and right, respectively. (**g**) A horizontal incision was performed in the basal, external and central part of the mouse ear and the external mouse ear skin layer was smoothly detached from the cartilage with a thin forceps. (**h**) Sponges were placed in the incision. (**i**) The gelatin sponge was introduced inside the hole, between the external mouse skin layer and the cartilage. A suture point was made to close the skin incision. (**j**) The same procedure was repeated in the other mouse ear, using always sponges with the same experimental condition in the same mouse.

The ear gelatin sponge assay was developed using small cylindrical pieces of compressed gelatin sponge populated with B16F10Luc+ tumor cells (Fig. 1 and Supplemental Movie SM1–2). A coat of interstitial type I collagen was used to confine tumor cells within this biomaterial (Fig. 1a–f and Supplemental Movie SM1). The sponges were next implanted between the external ear skin layer and the cartilage of mouse ears (Fig. 1g–j and Supplemental Movie SM2). In a first assay, tumor growth and draining LN (Supplemental Fig. SF1) colonization were compared after intradermal injection of B16F10Luc+ tumor cells (5 × 10^5^/50 μL) and after implantation of tumor cell-populated sponge (5 × 10^5^/sponge) into both ears. We first compared the bioavailability of D-luciferin in mice intradermally injected with 5 × 10^5^ tumor cells into ears and in mice implanted with sponges soaked with the same number of cells at day 0 (Supplemental Fig. SF2). After intraperitoneal (i.p.) injection of D-luciferin, the bioluminescent signal was detected in mice injected with tumor cells, but not in mice receiving cells-containing sponge. In sharp contrast, the local injection of D-luciferin inside the sponge led to bioluminescence detection (Supplemental Fig. SF2). This demonstrates that D-luciferin does not access to tumor cells embedded in the sponge and covered by collagen immediately after implantation (day 0). However, 2 days later bioluminescent signals were recorded in mice implanted with sponges.

**Figure 2:**
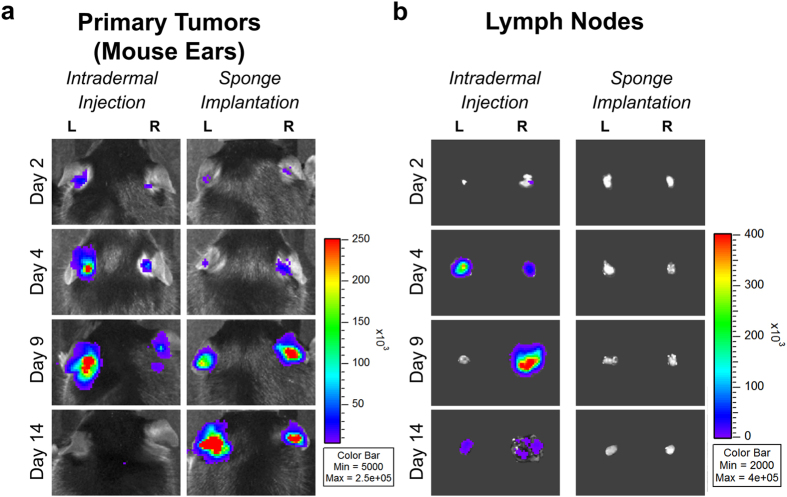
Comparison between the mouse ear sponge assay and the intradermal injection of tumor cells into mouse ears. The B16F10Luc+ tumor cell suspension (5 × 10^5^ cells/50 μl) was directly injected between the two ear skin layers of C57BL/6J mice (Intradermal Injection) or added on a cylindrical piece of sponge and then implanted in mouse ears (Sponge Implantation). (**a**) At different time points (Day 2, 4, 9 and 14) bioluminescence in mouse ears was recorded *in vivo* with a Xenogen system. (**b**) The draining sentinel LNs were dissected and their bioluminescence was visualized *ex vivo* over time. Numeric values displayed in the color scale for radiance are expressed in photons/sec/cm^2^. “L” represents left ears/LNs and “R” represents right ears/LNs, n = 8 sponges or LNs per group (4 mice/group).

After 2, 4, 9, and 14 days following intradermal tumor cell injections, Xenogen acquisitions in both ears were heterogeneous (Fig. 2a). It is worth mentioning that at Day 14 post-injection, the cells can be completely drained from the ear, with no bioluminescence signal detected in the ear (Fig. 2a). In sharp contrast, sponge implantations led to more homogenous bioluminescent signals, which increased over time (Fig. 2a). After intradermal cell injections, tumor cells were detected in sentinel LNs from Day 2 to Day 14 post-injection as assessed by the presence of bioluminescent signal through Xenogen acquisitions (Fig. 2b). A lower LN bioluminescence observed at Day 14 was associated with the spreading of metastatic cells into the mouse neck (Fig. 2b). By contrast, LN colonization by tumor cells did not occur within 14 days post-implantation in mice implanted with sponges (Fig. 2b). These data clearly show that the intradermal injection of tumor cells led to a rapid drainage of cells to LNs. However, tumor cell encapsulation in gelatin sponges led to local tumor growth in the ear without metastatic spreading within the two first weeks, allowing the study of pre-metastatic stages in sentinel LNs.

### Primary tumor growth and LN colonization at week 2 and 4 post-sponge implantation

**Figure 3:**
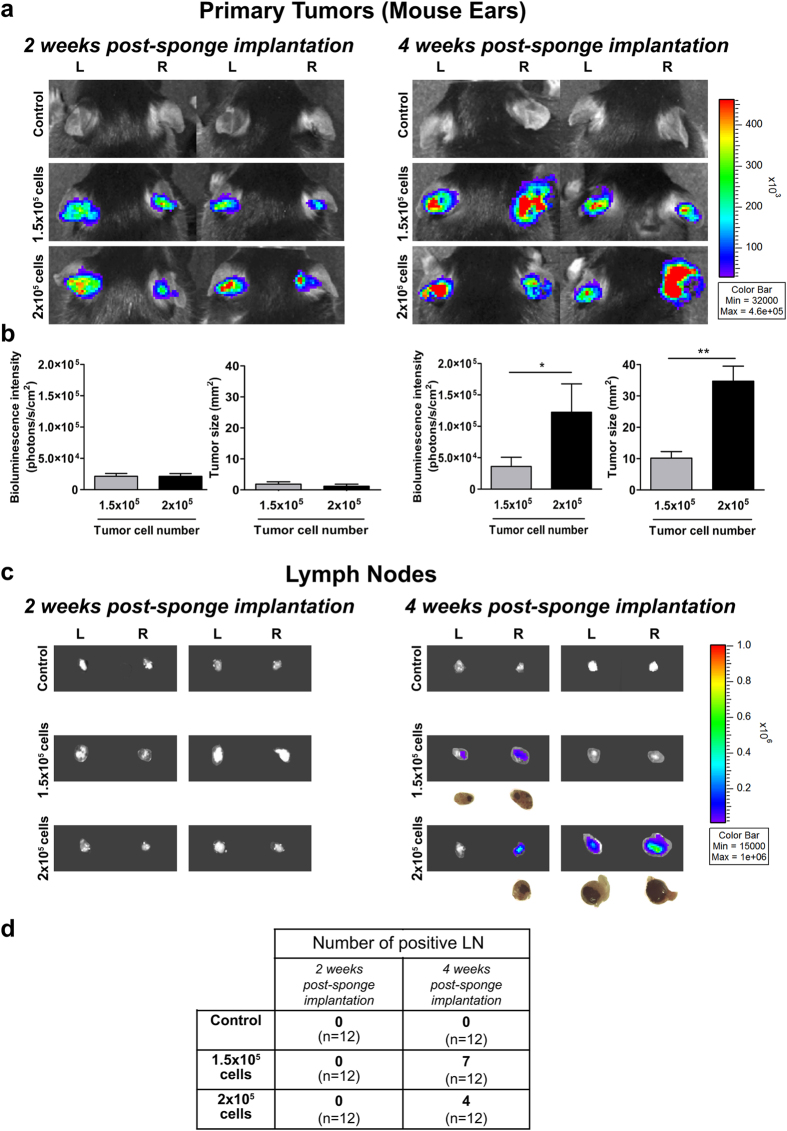
The mouse ear sponge model recapitulates the primary tumor growth, pre-metastatic and metastatic stages in LNs. Sponges soaked with serum-free DMEM medium (control condition without tumor cells), 1.5 × 10^5^ or 2 × 10^5^ B16F10Luc+ cells diluted in serum-free DMEM medium, were implanted into both C57BL/6J mouse ears and mice were kept for 2 or 4 weeks. (**a**) Representative pictures showing the *in vivo* bioluminescent signals of the primary tumors developed in ears, at 2 (left panels) and 4 (right panels) weeks post-sponge insertion. Numeric values depicted in the color scale for the radiance are expressed in photons/sec/cm^2^. (**b**) Quantification of bioluminescent signal radiance expressed in photons/sec/cm^2^ and measures of tumor sizes from histological sections of ear sponges. Data are presented as mean ± SEM, Wilcoxon-Mann-Whitney significance test was used to compare the mean parameter values, *p < 0.05, **p < 0.01, ***p < 0.001, n = 12 sponges per group (6 mice/group). (**c**) Representative pictures of *ex vivo* bioluminescent signal of the draining sentinel LNs at week 2 (left panels) and 4 (right panels) post-sponge implantation. Numeric values displayed in the color bar for radiance are expressed in photons/sec/cm^2^. For positive LNs, a bright field picture of LN is shown below the bioluminescent images. (**d**) Table of positive LNs incidence at week 2 and 4 post-sponge insertion. “L” represents left mouse ear/LN and “R” refers to right mouse ear/LN, n = 12 LNs per group (6 mice/group).

All the following experiments were performed with gelatin sponges soaked with B16F10Luc+ tumor cells or control medium, and tumor growth was analyzed after 2 and 4 weeks post-implantation. It is worth noting that sponges containing more than 2 × 10^5^ tumor cells generated necrotic primary tumors within 3 weeks (data not shown). Furthermore, a low incidence of LN colonization at 4 weeks was seen when using a number of cells lower than 1.5 × 10^5^ (data not shown). The implantation of ear sponges populated with 1.5 × 10^5^ or 2 × 10^5^ B16F10Luc+ tumor cells led to similar bioluminescence after 2 weeks (around 2.5 × 10^4^ photons/s/cm^2^) (Fig. 3a,b). These data were further confirmed by tumor size quantifications from ear sponge histological sections (Fig. 3b). However, 4 weeks after sponge insertion, the bioluminescent signal reached a 3-fold higher value in ear implanted with sponges soaked with 2 × 10^5^ cells than with those populated with 1.5 × 10^5^ cells (Fig. 3a,b) (*p *< 0.05). Higher tumor size was also observed in those sponges soaked with 2 × 10^5^ tumor cells (Fig. 3b). Of note is the higher heterogeneity observed in primary tumors induced by the highest number of tumor cells due to necrosis. When bioluminescence was checked in the sentinel LNs, again no positive signal was detected after 2 weeks of ear sponge implantation (Fig. 3c). In sharp contrast, LN positivity appeared after 4 weeks in mice implanted with sponges containing either 1.5 × 10^5^ or 2 × 10^5^ cells (Fig. 3c). No statistical significance was reached for metastasis size within positive LNs resected from mice implanted with different number of tumor cells (*p *> 0.05). The incidence of LNs colonized by tumor cells ranged from 33 to 58% (Fig. 3d).

### Lymphatic vasculature in the primary tumor

**Figure 4:**
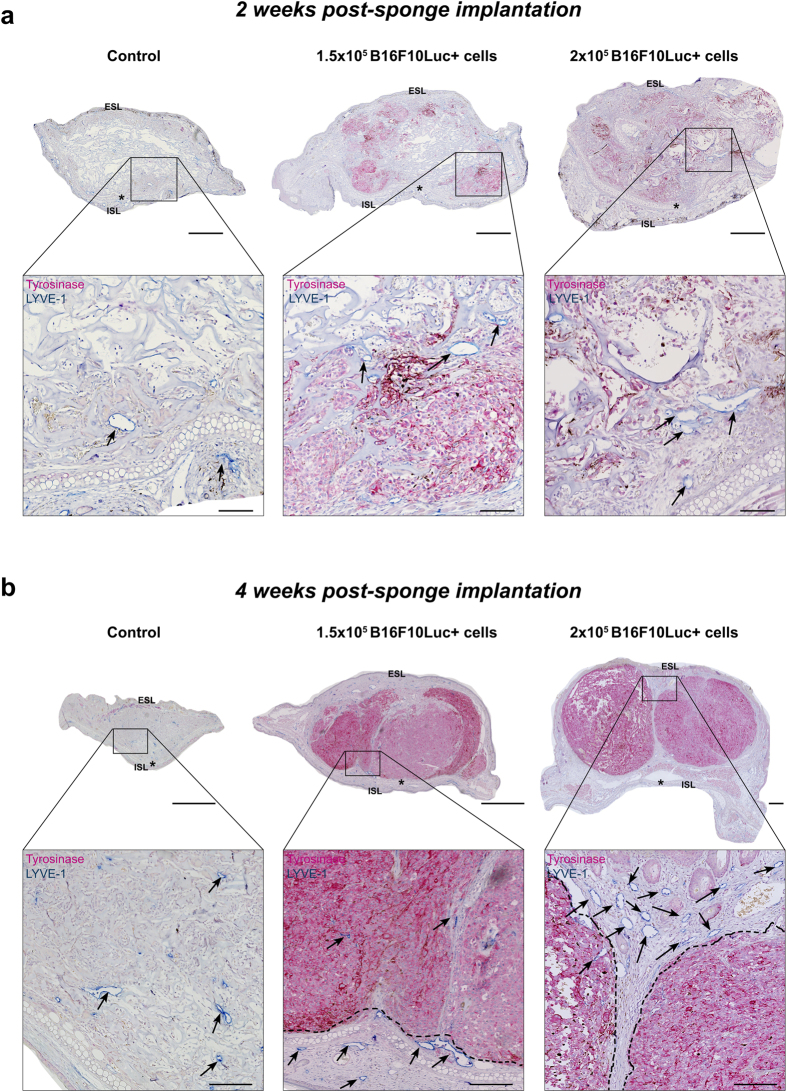
Immunohistochemical analyses of the primary tumors (mouse ears) after 2 and 4 weeks of sponge implantation. Control sponges devoid of tumor cells (control) and sponges populated with 1.5 × 10^5^ or 2 × 10^5^ B16F10Luc+ tumor cells were implanted in C57BL/6J mouse ears for 2 (**a**) or 4 (**b**) weeks. Lymphatic initial vessels (LYVE-1 positivity, blue staining) and tumor cells (tyrosinase expression, pink staining) were detected by immunohistochemistry, in control ears (sponges soaked with serum-free DMEM without tumor cells) and in ear-bearing primary tumors. Representative pictures are belonging to the central area of the ear sponge. Lower panels in “a” and “b” represent a higher magnification of the region delineated by the square in the upper pictures. Black arrows indicate some representative lymphatic vessels and the dotted black lines in “b” delineate the well-established tumor areas. ESL and ISL indicate the external and internal skin layers in mouse ears, respectively. Asterisk indicates the cartilage in mouse ears. Scale bars represent 500 and 100 μm in the low and high magnification pictures, respectively.

Immunohistochemical analyses were performed on the ear sponge sections to investigate the distribution of lymphatic vessels (LYVE-1+, blue staining) and tumor cells (tyrosinase+, pink staining) at 2 and 4 weeks after sponge implantation. The presence of tyrosinase positive tumor cells was seen in 100% of sponges soaked with tumor cells. In control ear sponge (devoid of tumor cells), initial lymphatic vessels were localized near to the ear border with few ones infiltrating the biomaterial (Fig. 4a). In tumor cell-populated sponges, lymphatic vessels were found deeper inside the biomaterial, intermingled with tumor cells (Fig. 4a). After 4 weeks, gelatin sponges were partially reabsorbed in all experimental groups. In control ears, most lymphatic vessels were again localized at the periphery of the tissue, but some of them were found deeper in the central part of the ear. At this time point, a compact primary tumor replaced the sponge initially implanted with tumor cells (Fig. 4b). Both peritumoral and intratumoral lymphatic vessels were detected.

**Figure 5:**
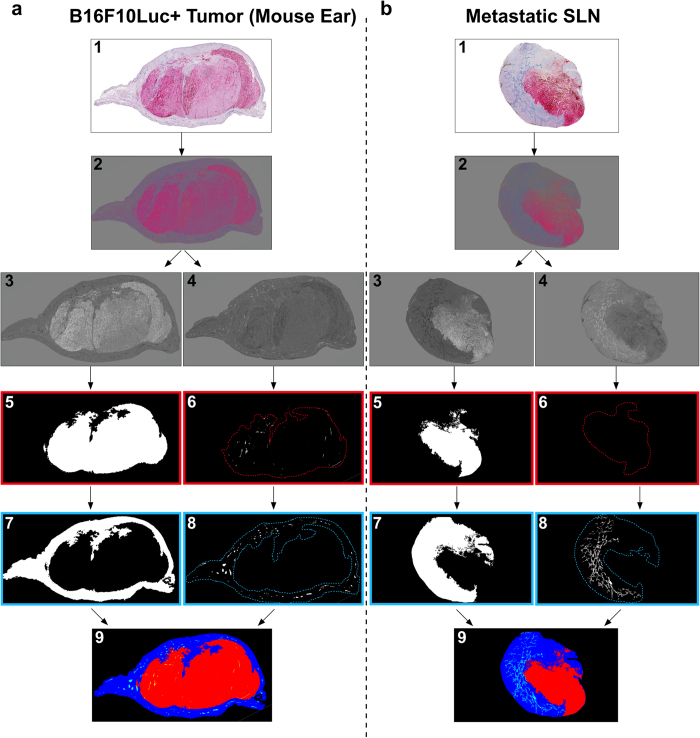
Computerized-assisted 2D image processing. Sections of the primary tumors (mouse ears) (**a**) and metastatic LNs (**b**) were immunostained for lymphatic vessels (LYVE-1 positivity in blue) and tumor cells (tyrosinase positivity in pink) (image 1), and subjected to image processing (images 2–9). (2) Contrast color enhancement using excess transformation. (3–4) Color image decomposition into its grey level components corresponding to (3) tumor (red component) and (4) lymphatic vessels (blue component). (5, 7) Automatic binarization of the (5) tumor and the (7) peritumoral region. (6, 8) Automatic binarization of (6) intratumoral vessels (confined inside the red dotted lines), and (8) peritumoral vessels (confined inside the blue dotted lines). (9) Color image compiling all the previous detected structures (peritumoral area in blue, tumor area in red, peritumoral vessels in light blue and intratumoral vessels in yellow).

**Figure 6:**
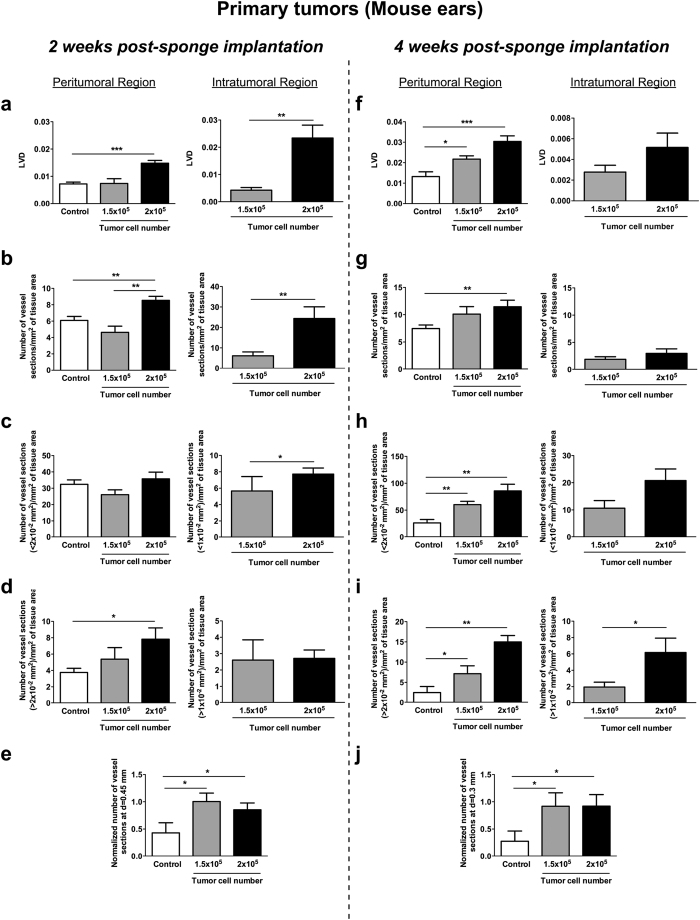
Computerized-assisted quantifications of the lymphatic vasculature in the primary tumors. Sponges devoid of tumor cells (controls) and sponges populated with 1.5 × 10^5^ or 2 × 10^5^ B16F10Luc+ tumor cells were implanted in the mouse ears for 2 (**a**–**e**) or 4 (**f**–**j**) weeks. Different parameters were measured on peritumoral and intratumoral areas. (**a**,**f**) The lymphatic vessel density (LVD). (**b**,**g**) The number of vessel sections per mm^2^ of specific tissue area. (**c**,**h**) The number of vessel sections smaller than a specific size (<2 × 10^−2^ mm^2^ or <1 × 10^−2^ mm^2^ for peritumoral or intratumoral, respectively). (**d**,**i**) The number of vessel sections larger than a specific size (>2 × 10^−2^ mm^2^ or >1 × 10^−2^ mm^2^ for peritumoral or intratumoral, respectively). (**e**,**j**) The normalized number of vessel sections at a distance of 0.45 mm or 0.3 mm from the ear border after 2 weeks or 4 weeks of sponge insertion. Results are expressed as mean ± SEM, and Wilcoxon-Mann-Whitney significance test was used to compare the mean parameter values, *p < 0.05; **p < 0.01; ***p < 0.001, n = 12 sponges per group (6 mice/group).

Computerized-assisted image processing and quantifications of lymphatic vessels were performed on whole scanned immunolabeled histological sections. During the image processing, original color images of primary tumors (Fig. 5a) and LNs (Fig. 5b) were binarized, allowing thereafter to delineate different regions of interest (tumor, peritumoral area and lymphatic vessels). All the delineated structures were gathered in a reconstructed picture (lower panels in Fig. 5). For quantification in primary tumors, peritumoral and intratumoral areas were always distinguished. For comparison purposes, quantifications performed on control sections and in peritumoral areas were considered together, since both of them were devoid of tumor cells. Vessel quantification in sponges implanted for 2 weeks (Fig. 6a–e) revealed that the lymphatic vessel density (LVD) and the number of vessel sections per mm^2^ of tissue area were twice as high in the peritumoral regions of sponges populated with 2 × 10^5^ B16F10Luc+ tumor cells, compared to control sponges or those populated with fewer tumor cells (*p *< 0.0003 *versus* control) (Fig. 6a,b). Then, the size of each vessel was calculated and histograms with the number of peritumoral or intratumoral vessels *versus* the vessel size were compared. Due to the wide range of vessel size in these distributions, percentiles were calculated and the cut off was applied at a value of 75% of this distribution. It corresponded to 2 × 10^−2^ mm^2^ and 1 × 10^−2^ mm^2^ for peritumoral and intratumoral vessel size, respectively. In those bigger tumors, the number of vessels with a size >2 × 10^−2^ mm^2^ was significantly enhanced, while no difference was seen regarding smaller vessels (<2 × 10^−2^ mm^2^) (Fig. 6c,d). The intratumoral LVD and the number of intratumoral vessels were also drastically increased in sponges populated with the highest number of cells (Fig. 6a,b). In the intratumoral area, vessels displaying a smaller size (<1 × 10^−2^ mm^2^) were detected slightly more frequently (Fig. 6c,d). The analysis of lymphatic vessel spatial distribution revealed that a twice-higher vessel number was detected at 0.45 mm from the ear border in sponges populated with tumor cells (Fig. 6e). From this specific distance, statistically significant differences were observed between control and experimental conditions. This data reflects the recruitment of lymphatic vessels inside ear sponges.

Quantifications at week 4 post-implantation (Fig. 6f–j) revealed a huge peritumoral lymphangiogenic response in sponges populated with 1.5 × 10^5^ and 2 × 10^5^ tumor cells. The peritumoral LVD and the number of peritumoral lymphatic vessels were both increased in those experimental groups, with peritumoral LVD being almost proportional to the number of cells used (Fig. 6f,g). Numbers of small peritumoral vessels (sections <2 × 10^−2^ mm^2^) and larger peritumoral vessels (sections >2 × 10^−2^ mm^2^) were enhanced in tumor-bearing mice and again was related to the number of tumor cells used (Fig. 6h,i). Regarding the intratumoral lymphangiogenesis, a lower intratumoral LVD and a lower lymphatic vessel number were detected in both groups of tumor-bearing mice, likely due to rapid tumor growth (Fig. 6f,g). Although no significant difference in the number of smaller vessels was detected, a 3-fold enhancement of the larger vessel number (sections >1 × 10^−2^ mm^2^) was observed in tumors induced by 2 × 10^5^ tumor cells (Fig. 6h,i). The analysis of lymphatic vessel spatial distribution revealed a huge lymphatic vessel migration towards the central area of the mouse ears. Indeed, the number of vessels found at a distance of 0.3 mm from the ear border was 4-fold higher in sponges populated with tumor cells (Fig. 6j). From this specific distance, statistically significant differences were observed between control and experimental conditions.

### Lymphatic vasculature and metastases in draining sentinel LNs

**Figure 7:**
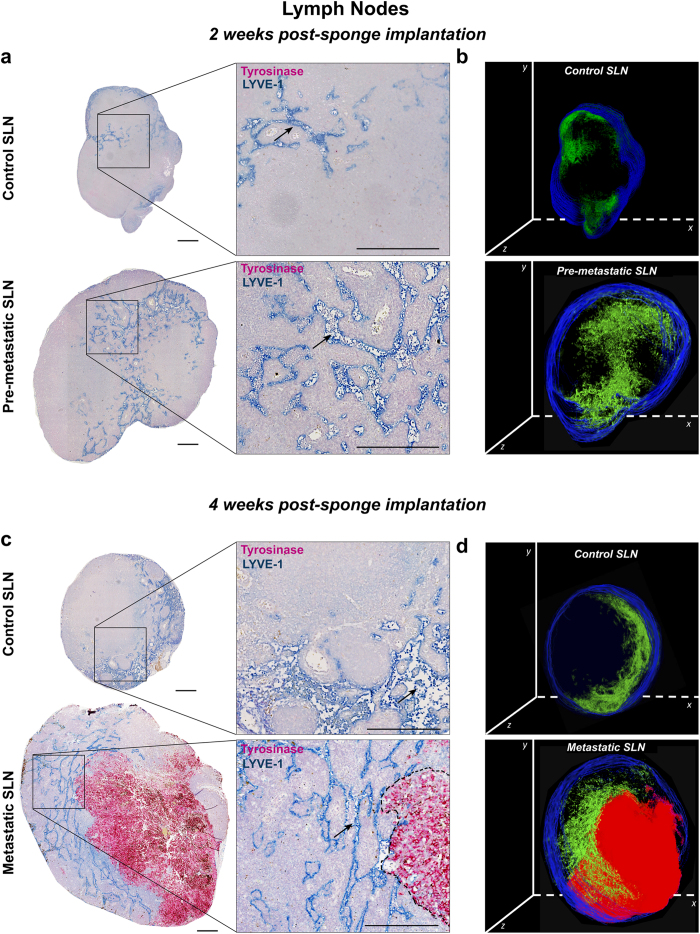
Immunostainings of lymphatic vessels and tumor cells in the draining sentinel LNs. Draining sentinel LNs were resected from mice with either control ear sponges or sponges populated with B16F10Luc+ tumor cells. (**a**) Representative sections, belonging to the central LN region, are shown for control SLN and pre-metastatic SLN after 2 weeks of sponge insertion. (**b**) 3D reconstruction of the representative control and pre-metastatic SLN shown in (**a**), with lymphatic vessels in green and tissue border in blue. (**c**) Representative sections of control SLN and metastatic SLN, belonging to the central LN region, after 4 weeks of sponge implantation. (**d**) 3D reconstruction of the representative control and metastatic SLN shown in (**c**), with lymphatic vessels in green, tumor mass in red and tissue border in blue. Right panels in (**a**) and (**c**) represent higher magnification pictures of areas delineated by the square. Immunostained lymphatic vessels (LYVE-1 positive) appear in blue and tumor cells (tyrosinase positive) in pink. Black arrows indicate representative lymphatic vessels and the black dotted line delineate the tumor area. Scale bar represents 250 μm. For 3D reconstructions of SLN, around 40 sections were stacked, with a final z-stack thickness of ∼ 200 μm.

**Figure 8:**
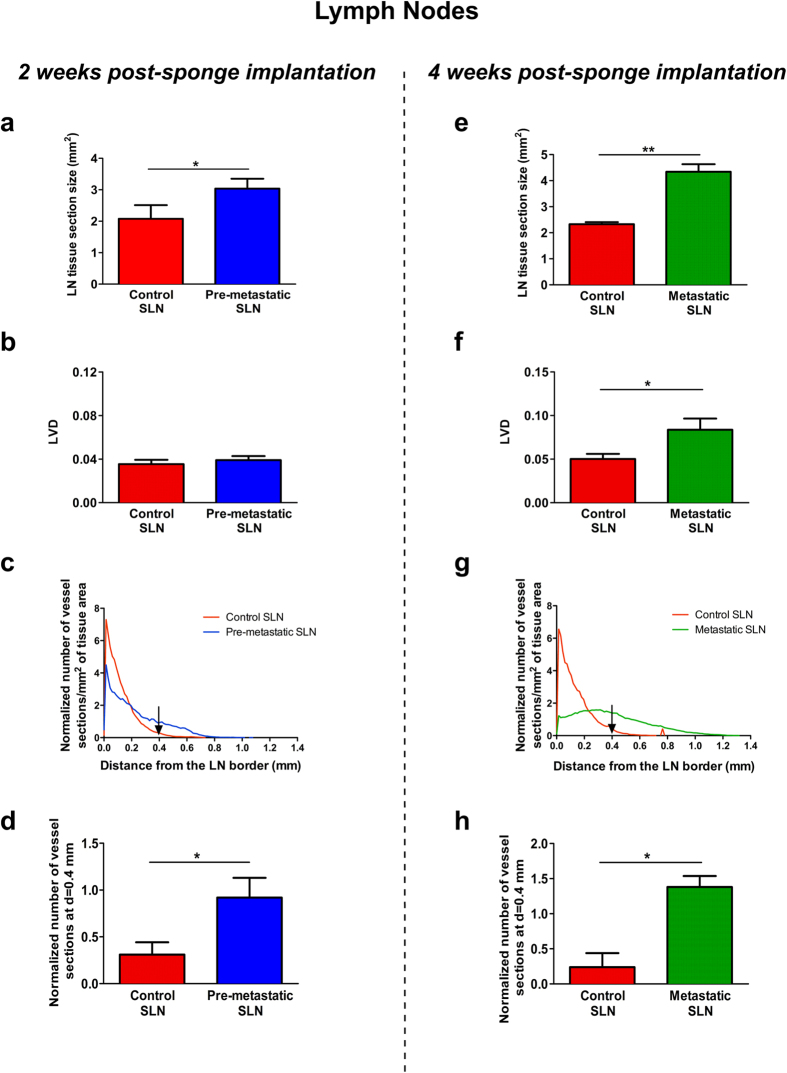
Computerized-assisted quantifications of lymphatic vasculature in the draining sentinel LNs. Quantitative analyses were performed on immunolabeled control and pre-metastatic SLN sections from LNs resected after 2 weeks of sponge implantation (**a**–**d**), and on immunolabeled control and metastatic SLN dissected after 4 weeks (**e**–**h**) of sponge insertion. (**a**,**e**) The LN tissue section size (mm^2^) in control SLN (red bar), pre-metastatic SLN (blue bar) and metastatic SLN (green bar). (**b**,**f**) The lymphatic vessel density (LVD). (**c**,**g**) The lymphatic vessels’ spatial distribution curves from the LN border (distance = 0). (**d**,**h**) The histogram corresponds to the number of lymphatic vessel sections at a distance of 0.4 mm from the border of LN. Results are expressed as mean ± SEM, and Wilcoxon-Mann-Whitney significance test was used to compare the averaged parameter values measured for the different groups, *p < 0.05, n = 12 LNs per group (6 mice/group).

Lymphatic vessels and B16F10Luc+ tumor cells in LNs resected from mice after 2 and 4 weeks of sponge implantation were detected by double immunolabeling for LYVE-1 and tyrosinase, respectively. Importantly, sentinel LNs issued from mice implanted with gelatin sponges soaked with tumor cells for 2 weeks were negative for tyrosinase staining, revealing the absence of metastatic tumor cells (Fig. 7a). These data are in line with the absence of bioluminescent signal detected through the Xenogen acquisitions (Fig. 3b). It is worth noting that the negative sentinel LNs, considered as pre-metastatic LNs, displayed a larger size than control LNs and lymphatic vessels extending to the central LN region (Fig. 7a). The lymphatic vasculature of control sentinel LNs (issued from non-tumor-bearing mice) was restricted to the cortex as underlined in 3D reconstruction (Fig. 7b). In sharp contrast, lymphatic vessels were enlarged and distributed throughout the paracortex and medullar area in pre-metastatic LNs (Fig. 7a,b). At week 4 post-implantation, around 50% of analyzed LNs were colonized by tumor cells, showing positivity for tyrosinase, and they were classified as metastatic sentinel LNs. The lymphatic vasculature was denser in the medulla surrounding the tumor mass (Fig. 7c,d), suggesting drastic lymphangiogenesis and migration of lymphatic vessels from LN border to the center. Interestingly, an increase of the whole LN section size was observed in pre-metastatic and metastatic LNs (Fig. 8a,e).

An in-depth characterization of LN lymphatic vasculature, after 2 (Fig. 8b–d) and 4 (Fig. 8f–h) weeks of sponge insertion was conducted using our computerized-assisted method described above. While the LVD after 2 weeks was similar in control and pre-metastatic LNs (Fig. 8b), the spatial distribution of lymphatic vessels differed in control and pre-metastatic LNs (Fig. 8c). Indeed, most of lymphatic vessels were confined near to the LN border in control LNs (between 0 and 0.2 mm) (Fig. 8c). In contrast, lymphatic vessels were found at a distance up to 0.8 mm from the border in pre-metastatic LNs (Fig. 8c). Interestingly, the normalized number of lymphatic vessel sections at a specific distance of 0.4 mm from the border was increased 3-fold in pre-metastatic LNs (Fig. 8d). Thus, despite the absence of tumor cells, the spatial distribution of lymphatic vessels in pre-metastatic LNs was clearly different to that observed in the control sentinel LNs. An increased LVD was found in metastatic LNs than in control and pre-metastatic LNs (Fig. 8f). The spatial distribution analysis revealed a deeper migration of lymphatic vessels towards LN center. The maximal distance (Lmax) reached by lymphatic vessels was 0.7 mm and 1.2 mm from the surface of control and metastatic LNs, respectively (Fig. 8g). At a distance of 0.4 mm from the LN border, the normalized number of vessel sections was increased 4.5-fold in metastatic LNs compared to control LNs (Fig. 8h).

## Discussion

There is an urgent need for well-established *in vivo* models to study the pre-metastatic niche in sentinel LNs, the major metastatic site for many solid cancer types such as melanoma and most carcinomas. Here we describe for the first time, a gelatin sponge-based model, which is suitable to analyze the lymphatic vasculature in a primary tumor and the pre-metastatic and metastatic lymphvascular niche in mice LNs. In this system, tumor cells confined to a biomaterial stimulate local and distant lymphangiogenesis potentially generating a fertile microenvironment in LNs that could facilitate their colonization by tumor cells. This robust and reliable model is easy to perform and is associated to tumor cell tracking by bioluminescence and to a computerized-assisted method of quantification on tissue sections for the in-depth characterization of the lymphatic vasculature. To set up our model, we used B16F10 melanoma cells since most cutaneous malignant melanomas in human patients metastasize first to LNs via lymphatic vessels.

The originality of our model relies on (i) the confinement of tumor cells in a gelatin sponge surrounded by a collagen matrix, and (ii) their implantation in mouse ears, a lymphatic-rich tissue. It is worth mentioning that the biomaterial used in our model has been previously used with other purposes. For instance, small pieces of gelatin sponges have been inserted in a rabbit liver tumor model of hepatic arterial chemoembolization^18^ or have been used as embolization agents for the treatment of the spontaneous rupture in hepatocellular carcinoma hemorrhage^19^. Other types of tumor or non-tumor cell encapsulation have been already used. They include at least the use of semipermeable membranes for the orthotopic implantation of pancreatic cancer cells^20^ and alginate or alginate/gelatine microcapsules^21^,^22^. Tumorspheres or cells embedded into matrigel are also widely used to confine tumor *in vivo*^17^,^23^.

It has been previously shown that the intradermal injection of tumor cells in mouse ears can lead to the direct drainage of cells to LNs^24^. Accordingly, the intradermal injection of melanoma cells led to a rapid LN colonization within 2 days, as assessed by *ex vivo* bioluminescence detection. The presence of tumor cells in LNs thus reflects the direct cell entry into lymphatic vessels during cell injection, rather than a real metastatic spreading. We consider that this type of cell inoculation in the ears is analogous to the so-called “hematogenous experimental metastasis” induced by the intravenous injection of tumor cells. In sharp contrast, in mice implanted with a tumor-populated sponge, no LN metastasis was detected during the 2 first weeks post-implantation, whereas tumors grew locally. This finding demonstrates that sponges soaked with tumor cells and embedded in a collagen matrix provide an appropriate scaffold for tumor growth and avoid the direct escape of tumor cells from primary tumors to LNs. Of great interest is the potential of this model to investigate the environment in the primary tumor and in LNs, both at pre-metastatic and metastatic stages.

In primary tumors, an intense lymphangiogenic response was induced by B16F10Luc+ cell populated gelatin sponges and occurred both within the primary tumor mass (intratumoral lymphangiogenesis) and in the tumor periphery (peritumoral lymphangiogenesis). This observation is in line with clinical data^25^,^26^,^27^. It is generally believed that only peritumoral vessels are functional and provide the major route for fluid and cell drainage from the primary tumor^28^,^29^,^30^. In support of this concept, in our system, peritumoral lymphatic vessels appeared dilated, tortuous in shape and were filled with cells (Supplemental Fig. SF3). In sharp contrast and again in line with clinical data^28^, intratumoral vessels were often small in caliber and appeared collapsed in histological tissue sections.

Besides the study of the primary tumor microenvironment, our system permits to analyze the changes occurring in the lymphvascular niche of LNs during pre-metastatic stages. Two weeks post-sponge implantation, while no tumor cells were detected in LNs, a significant enlargement and migration of lymphatic vessels from the cortex to the medullar LN area was observed. Importantly, LNs in pre-metastatic stage exhibited an expansion in size and cellularity, reflecting important tissue remodeling even before tumor cell arrival. The secretion of pro-inflammatory cytokines by tumor cells in the primary tumor^31^,^32^, as well as the production of lymphangiogenic factors by tumor cells or immune cells (macrophages or B-cells)^33^,^34^ are well documented and contribute to important LN remodeling^35^,^36^. For instance, VEGF-A or VEGF-C overexpression in a murine model of skin carcinogenesis is associated with increased lymphangiogenesis in the primary tumor and in the draining LNs^37^. The current mechanisms proposed to explain how tumor cells gain access to the lymphatic vasculature and establish sentinel LN metastasis include: (i) the growth and remodeling of the lymphatic vasculature in the tumor microenvironment; (ii) the increased fluid drainage from tumor tissue, which provokes the passive transport of tumor cells; and (iii) the guidance of tumor cell migration towards lymphatic vessels by chemokines such as CCL21 and CXCL12, produced by activated LEC^28^,^38^,^39^. Regarding metastatic LNs, it is worth mentioning that a robust lymphatic network was observed around the tumor mass, while no intratumoral lymphatic vessels were detected. The well pre-established lymphvascular network thus potentially provides an adequate niche for the survival and growth of metastatic tumor cells.

In order to study LN metastasis, Harrell and colleagues carried out the direct injection of B16F10 melanoma cells in the hind footpad of syngeneic C57BL6 mice^40^. This model differs from ours because they used a strategy likely based on the so-called “experimental metastasis”, without the spatial confinement of tumor cells inside a biomaterial. In such study, slow growing primary tumors were generated without lymphangiogenic response. In LNs during pre-metastatic stages, they reported an increase in the lymphatic vessel area, without considering the size of LNs, which can be affected by the presence of a primary tumor, as shown here. Our results point out the importance of considering lymphatic vessel density (vessel area divided by LN surface) rather than lymphatic vessel area when investigating the lymphangiogenic response in draining LNs. In this context, most of the studies performed on lymphangiogenesis are reporting LVD data^28^,^41^,^42^.

The herein proposed model is a rapid and reproducible model associated with a robust method of quantification. The collagen-coated sponges are easy to implant and the assay neither require considerable technical skills, nor a complex and invasive surgical procedure. Importantly, in contrast to numerous models of tumor metastasis in which mice need to be sacrificed because of the large size of the primary tumor^9^, here no tumor resection is required to allow the study of metastatic spreading. Notably, it facilitates the analysis of the cross-talk established between tumor cells and the lymphatic vasculature in primary tumors and in LNs. Additionally, in contrast to several *in vivo* mouse assays in which the quantification of lymphatic vessels is tedious, the computerized-assisted method of quantification used here is rapid and accurate, allowing the deep characterization of the lymphatic vasculature. Furthermore, this biological model, together with the image processing tools offers the possibility to perform 3D reconstructions of primary tumors, pre-metastatic, and metastatic LNs (Fig. 7). It is worth noting that the ear sponge assay is suitable for testing the *in vivo* lymphangiogenic potency of tumor cells or diverse factors of interest. Indeed, the activity of lymphangiogenic agonists or antagonists can be assessed in wild type mice or transgenic mice. One advantage over existing methods used to assess angiogenesis *in vivo* in mice or rats^43^,^44^, is the unique possibility to concomitantly study both angiogenic and lymphangiogenic responses^45^,^46^. On the other hand, the small volume required in this biomaterial-based *in vivo* model to soak the sponge with a tested compound makes it ideal in the case of drug limitation. Moreover, compounds with low stability can be easily re-injected around the sponge. Therefore, this novel model offers numerous possibilities to address current crucial issues in the field of cancer and pathological lymphangiogenesis.

Our study presents a new sponge-based *in vivo* model designed to study the lymphatic vascular network in primary tumors and to characterize the lymphvascular niche in sentinel pre-metastatic and metastatic LNs. The ear sponge lymphangiogenic assay offers a simple, reliable, relevant and quantitative *in vivo* assay for lymphangiogenesis assessment. This approach is expected to facilitate and optimize studies of tumor-associated lymphangiogenesis *in vivo*, holds promise for unraveling the mechanisms underlying pre-metastatic niche formation in LNs and will be useful for the development of novel and more efficient drugs targeting tumor-induced lymphangiogenesis.

## Methods

### Cell lines

Murine B16F10Luc+ melanoma cells expressing luciferase (Luc+) (Caliper Life Sciences, Hopkinton, MA) were used in this study. Cells were cultured in Dulbecco’s modified Eagle’s (DMEM; Gibco Invitrogen Corporation, Paisley, United Kingdom) supplemented with 1% glutamine, 1% penicillin/streptomycin and 10% Fetal Bovine Serum (FBS) in standard conditions (humidified atmosphere containing 5% CO_2_ at 37 °C). To ensure the presence of luciferase reporter, cells were maintained under selective pressure with G418 (gentamicin) at a final concentration of 1mg/ml. Before each *in vivo* assay, luciferase activity was checked by Dual Luciferase Reporter Kit (150 ng∙mL^−1^; Promega, Leiden, Netherlands) according to the manufacturer’s specifications.

### Direct intradermal tumor cell injections into mouse ears

For the direct injection of B16F10Luc+ tumor cells, mice were anesthetized with ketamine hydrochloride (100 mg/kg body weight) and xylazine (10 mg/kg body weight), and intradermal injection of DMEM (50 μL) containing 5 × 10^5^ B16F10Luc+ cells was performed as previously described by Bobek *et al*.^24^. After 2, 4, 9 and 14 days, mice were injected intraperitoneally with 75 mg/kg D-luciferin (Caliper Life Sciences), anesthetized with isoflurane and *in vivo* bioluminescence was recovered as described below. All animal experiments were performed in strict compliance with the European Communities Council Directive 2010/63/EU and the Belgium legislation for the animal experimentation. The Local Animal Ethics Committee at the University of Liège approved the ethical and legal aspect of our experimental protocol (13/1522) before the starting of the research. Researchers working with animals received a specific training to reach the Category C from the Federation of European Laboratory Animal Science Associations (FELASA) before animal manipulation. Animals were housed within the accredited animal facility of GIGA (University of Liège, Belgium).

### Gelatin sponge preparation

A sterile compressed gelatin sponge (GELFOAM, Pfizer, Puurs, Belgium) was cut into small cylindrical pieces (around 3 mm^3^) (Fig. 1a,b and Supplemental Movie SM1). After checking Luciferase expression by bioluminescence in B16F10Luc+ tumor cells (Fig. 1c), the sponges were soaked with either tumor cell suspensions (1 × 10^5^, 1.5 × 10^5^, 2 × 10^5^, 3 × 10^5^ or 5 × 10^5^ cells diluted in serum-free DMEM) or control medium (serum-free DMEM without tumor cells) by adding a 20-μl drop on top of the sponge (Fig. 1d and Supplemental Movie SM1). Sponges were incubated for a period of 30 min at 37 °C, and then they were rapidly embedded (3 baths by immersion) in a cold interstitial type I collagen solution [7.5 volumes of Collagen R at 2 mg/ml (SERVA Electrophoresis, Heidelberg, Germany), 1 volume of 10x Hanks´ Balanced Salt Solution (HBSS; Gibco), 1.5 volume of NaHCO_3_ 186 mM and 1 volume of NaOH 1 M to adjust the pH to 7.4, according to modification of HBSS color from yellow to purple] making a capsule around the sponge. Sponges were immediately transferred into new wells and re-incubated 30 min at 37 °C to allow complete collagen gel polymerization and ensure that the added cells remained inside the sponges (Fig. 1e and Supplemental Movie SM1). The bioluminescence of the soaked sponges was measured next (Fig. 1f).

### Gelatin sponge implantation into mouse ears

C57BL/6 mice (Charles River, Saint-Germain-Nuelles, France) aged 6-to-8-weeks old were anesthetized as described above, and a small horizontal incision was performed in the basal, external, and central part of the mouse ear. The external mouse ear skin layer was smoothly detached from the cartilage making a hole of 5 mm^2^ (Fig. 1g and Supplemental Movie SM2) and the gelatin sponge was introduced deeper in the hole (Fig. 1h and Supplemental Movie SM2). Once the sponge was placed inside the ear, a suture point was made (Fig. 1i and Supplemental Movie SM2). The same procedure was repeated in the other mouse ear (Fig. 1j and Supplemental Movie SM2) and sponges implanted in the same animal were always soaked with the same number of cells. After surgery, the animal was left on a warming plate (34 °C) to allow the mouse to recover from anesthesia. The two sponges were considered as independent entities.

### *In vivo* imaging

At different time points after tumor cell injections, and 2 or 4 weeks after sponge implantation, the mice were injected intraperitoneally with 75 mg/kg D-luciferin (Caliper Life Sciences) and anesthetized with isoflurane. The animals’ ears were imaged 12 min post-injection for maximum bioluminescence signal (Xenogen IVIS 200 Imaging System, Caliper Life Sciences, Hopkinton, MA). For quantification, Living Image software (Caliper Life Sciences) was used according to the manufacturer’s recommendations. Then, the mice were sacrificed by cervical dislocation, in accordance with the guidelines of the local animal ethical committee. The ears were cut and sentinel LNs were resected for immediately measuring their bioluminescence as described above. They were next visualized and photographed with a Leica modular routine stereo microscope coupled with a Full-HD Leica IC80 HD camera (Leica Microsystems, Wetzlar, Germany).

### Immunohistochemistry

After ear imaging and excision, the skin was cut around the sponge. For visible stainings, the sponges and LNs were fixed in 4% formalin (overnight or for 4 h, respectively) followed by overnight paraffin embedding. Before the immunohistochemistry, sections of 5 μm were left at 60 °C overnight, deparaffinized and hydrated. For neoformed and initial lymphatic vasculature detection, sections were autoclaved for 11 min at 126 °C in citrate solution, washed with distilled water (2 times × 5 min), incubated with a serum-free protein block solution (10 min) (Dako, Glostrup, Denmark) and next incubated with LYVE-1 antibody (R&D Systems, Minneapolis, MN) for 2 h (dilution 1:100 in Dako diluent, at room temperature). After washing in PBS, staining was revealed with the Ferangi Blue chromogen kit (BioCare Medical, Concord, United States). To detect tumor cells (B16F10 melanoma cells expressing tyrosinase protein), an antibody against tyrosinase (Abcam, Cambridge, UK; rabbit anti-human antibody diluted 1:500 in Dako diluent, overnight at 4 °C) was used after blocking the endogenous peroxidase (3% of H_2_O_2_) for 10 min and incubation of samples with normal goat serum 100% (CER Groupe, Marloie, Belgium) for 30 min. The signal was revealed after 30 min of incubation with PowerVision anti-rabbit/AP (Immunologic, Duiven, Netherlands) followed by Permanent Red substrate chromogen (Dako). Samples were counterstained with hematoxylin/eosin, washed in water, dehydrated in graded alcohols and mounted with EUKITT. In Supplemental Fig. SF4 is depicted how tumor-bearing ears (a) and LNs (b) were sectioned and the position of the representative samples shown in Figs 4 and 7.

### Computerized-assisted image processing

Images from whole immunolabeled tissues were captured with a NanoZoomer 2. OHT scanner (Hamamatsu, Mont-Saint-Guibert, Belgium). Histological section images were registered in full color red, green and blue (RGB). On those images, neoformed and initial lymphatic vessels (LYVE-1 positivity) and tumor cells (tyrosinase positivity) were visualized as blue and pink colors, respectively. The original algorithm of image analysis was implemented to automatically perform two successive steps (image processing and measurements) by using the image analysis toolbox of MATLAB 8.3 (R2014a) software (Mathworks, Inc). At first, image processing was applied to eliminate noise and thresholding transformation was carried out to obtain binary images. By the last operation, all pixels belonging to objects of interest (vascular network or tumor cells) take the value 1 and those of the background the value 0^47^,^48^ (see Supplemental Methods for details). When automatic feature detection was not accurate, the threshold was adapted manually. For 3D image reconstructions, LNs were always fully sectioned in 5 μm slices, and one sample over 10 was selected for sequential immunohistochemistry. Histological sections were processed to obtain 2D binarized pictures, re-oriented with the same spatial orientation and stacked using ImageJ software. Around 40 sections were stacked to provide a 3D reconstruction, with a final z-stack thickness of ∼200 μm.

### Computerized-assisted quantification of the vascular network in primary tumors and LNs

For vasculature characterization and quantification, we measured the following parameters: (i) lymphatic vessel density (LVD), defined as the area occupied by lymphatic vessel divided by the specific tissue area; (ii) number of vessel sections per mm^2^ of the specific tissue area; (iii) the number of vessel sections smaller or larger than a specific size per mm^2^ of the specific tissue area; (iv) spatial distribution of lymphatic vessels from the tissue border to the central tissue area. For the spatial distribution, the Euclidean distance between each pixel belonging to the vessels and the border of the tissue was determined. Then, the non-parametric Parzen method^49^ was used. This method is not based on any assumption about the distribution shape and provides a normalized distribution with an area under the curve equal to 1. All parameters described above were measured on each tissue section belonging from primary tumors (12 tissue sections taken at different levels from each complete sponge) or LNs (40 tissue sections taken at different levels from each whole LN). The resulting values and functions were averaged. Final results were expressed as the mean of the values obtained for each set of the different primary tumors or LNs analyzed. The two sponges/LNs from the same mouse were considered as independent entities.

In primary tumors, the measured parameters described below, were applied to the lymphatic vasculature present in the peritumoral (established around the tumor area) and intratumoral (inside the tumor mass) regions. In metastatic LNs (colonized by tumor cells), no lymphatic vessels were present inside tumors (intratumoral LVD = 0). For clarity reasons, only quantifications performed in the peritumoral regions are shown.

### Statistical analysis

For bioluminescence quantification in the mouse ears and LNs, data are presented as mean ± SEM (standard error of the mean) and *t*-test was applied using Prism 4.0 software (GraphPad, San Diego, CA). For computerized-assisted image analysis, statistical analysis was performed using the statistic toolbox of MATLAB 8.3 (R2014a) software. Results are expressed as mean ± SEM and a Wilcoxon-Mann-Whitney significance test was used to compare the averaged parameter values measured in the different experimental groups. “n” represents the number of sponges or LNs analyzed per group. Results were considered significant at *p *< 0.05.

## Additional Information

**How to cite this article**: García-Caballero, M. *et al*. Modeling pre-metastatic lymphvascular niche in the mouse ear sponge assay. *Sci. Rep.* **7**, 41494; doi: 10.1038/srep41494 (2017).

**Publisher's note:** Springer Nature remains neutral with regard to jurisdictional claims in published maps and institutional affiliations.

## Supplementary Material

Supplemental Movie SM1

Supplemental Movie SM2

Supplementary Data
