# Supplementary material for: Modeling pre-metastatic lymphvascular niche in the mouse ear sponge assay
Source: Sci Rep. 2017 Jan 27;7:41494. doi: 10.1038/srep41494 (PMC5270255; doi:10.1038/srep41494)
Supplement: Supplementary Data [file srep41494-s3.pdf]

Modeling pre-metastatic lymphvascular niche in the mouse ear sponge assay

Melissa García-Caballero[1,3*], Maureen Van de Velde[1,3], Silvia Blacher[1], Vincent Lambert[1], Cédric Balsat[1], Charlotte Erpicum[1], Tania Durré[1], Frédéric Kridelka[1,2], Agnès Noel[1]

[1] Laboratory of Tumor and Developmental Biology, GIGA-Cancer, University of Liège, Sart-Tilman, B-4000, Liège, Belgium
[2] Department of Obstetrics and Gynecology, CHU Liège, Sart-Tilman, B-4000, Liège, Belgium
[3] Co-first authors

SUPPLEMENTARY METHODS

1. Computerized-assisted image processing

Initially, in order to increase the contrast between the stained regions of the primary tumor or LN samples, and the surrounding tissue, the color excess transformation from the three RGB components (two times each component value minus the value of the others two components) was calculated. After splitting the resulting enhanced color image into its RGB components, each component was processed separately. The whole tissue section, which was well contrasted in relation with the background, was detected automatically from the blue component using an appropriate threshold. For red (tumor cells) and blue (lymphatic vessels) components, a low pass morphological filter ("close transformation") was applied to eliminate noise. Then, images were binarized using an automatic entropy threshold. Finally, an erosion filter was used to eliminate small artifacts, and the resulting image was used as marker for a geodesic reconstruction of the final binary image. To obtain the peritumoral region, the tumor area was substracted from the binary images of tissue section. Of note, the morphology of lymphatic vessels in primary tumors and in LN was different. Although they appeared as rounded and independent structures in tumors, they were tortuous and continuous in LN. In the first case, where the whole lumen boundaries were not completely detected they were completed manually. Then, the structure was automatically filled for quantifying the whole vessel section. The irregular and tortuous structure of lymphatic vessels in LN sections prevents the use of same procedure. In this case, blue staining corresponding to lymphatic vessels was automatically segmented. It is worth noting that the binary images resulting from the image process described above were systematically compared with the corresponding originals and in the cases that automatic feature detection was not accurate, the threshold was adapted manually.

Supplemental Fig. SF1

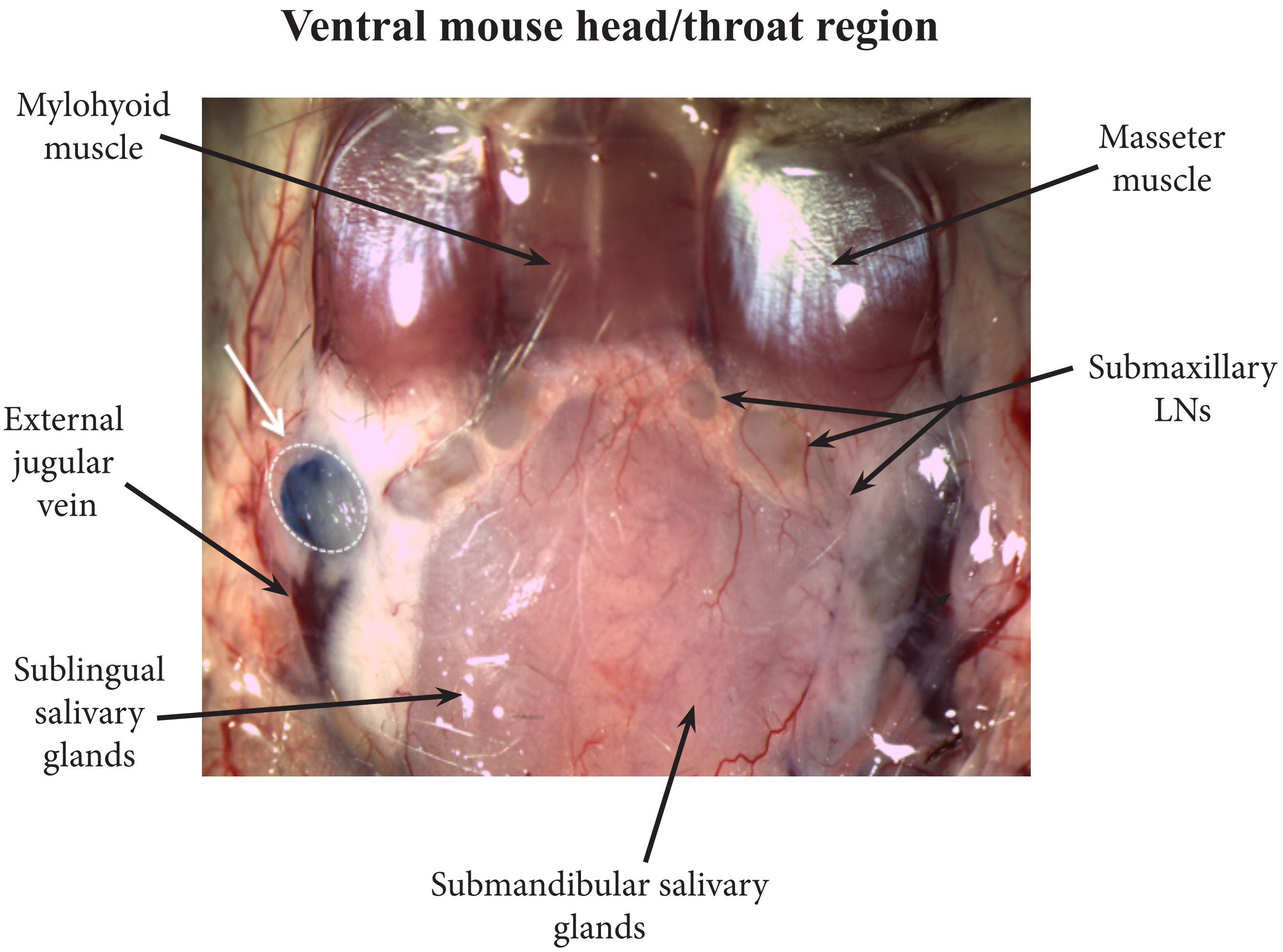

Supplemental Fig. SF1: Sentinel LN identification by Evans blue injections into the mouse ears. A total of 20 µL of Evans blue dye solution in PBS (6 mg/mL) was injected subcutaneously into the ear of anesthetized C57BL6 mice. Next, mice were sacrificed and sentinel LN were visualized by the presence of a blue coloration, indicated with a white arrow in the picture.

Supplemental Fig. SF2

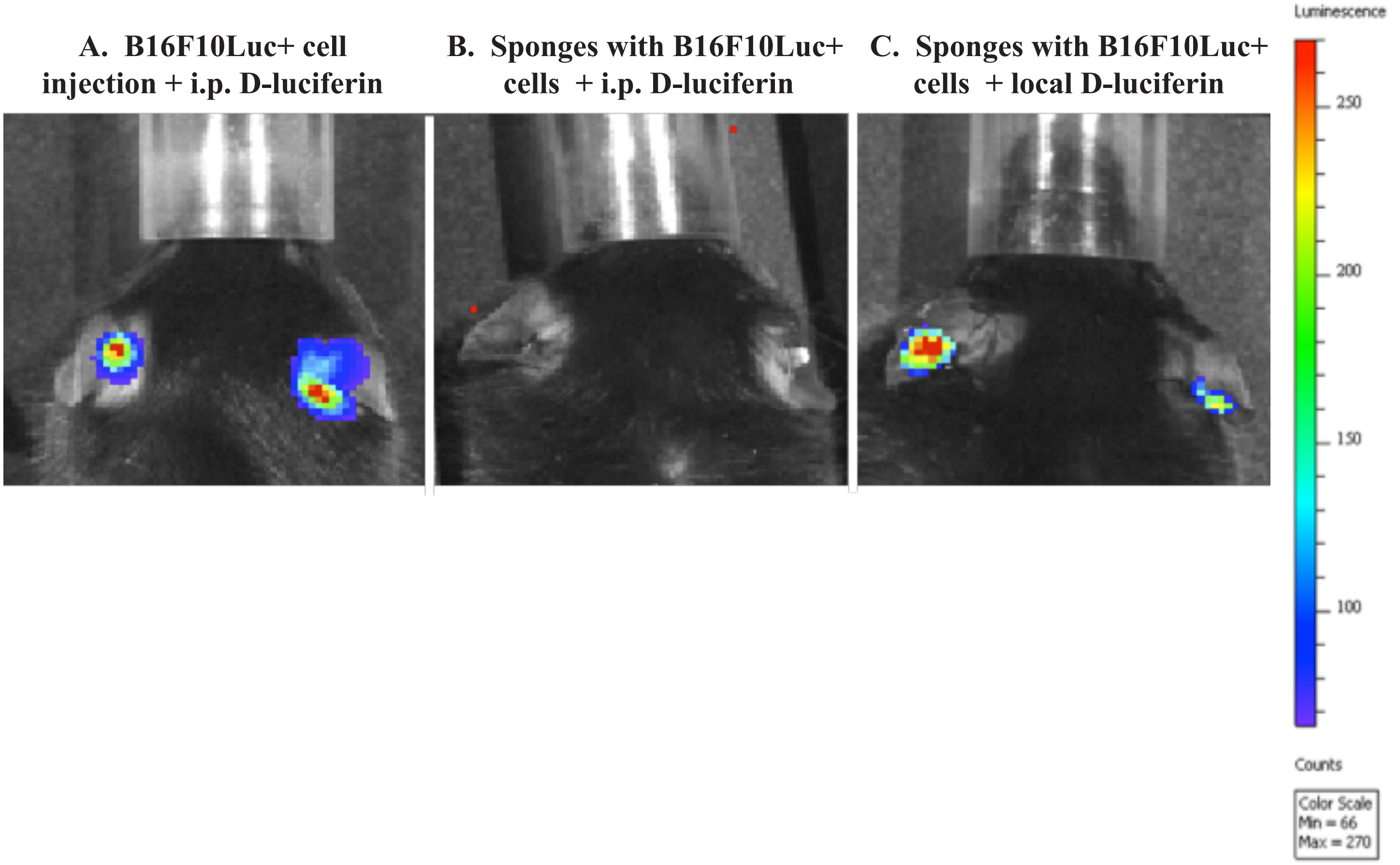

Supplemental Fig. SF2: Bioavailability of D-luciferin within the sponge. Xenogen acquisitions in mice with direct injections or sponge-implanted with tumor cells into the ears. Tumor cells (B16F10Luc+) were either directly injected into ear (A) or embedded in a sponge implanted into the ear (B, C). After i.p. D-luciferin injection, bioluminescent signal was recorded at day 0 when cells were directly injected in the ear (A), but not when soaked in a sponge (B). D-luciferin injection in the sponge containing cells led to bioluminescence detection (C).

Supplemental Fig. SF3

Primary Tumors (Mouse Ears)

4 weeks post-sponge implantation

1.5x10^5 B16F10Luc+ cells | **2x10^5 B16F10Luc+ cells**

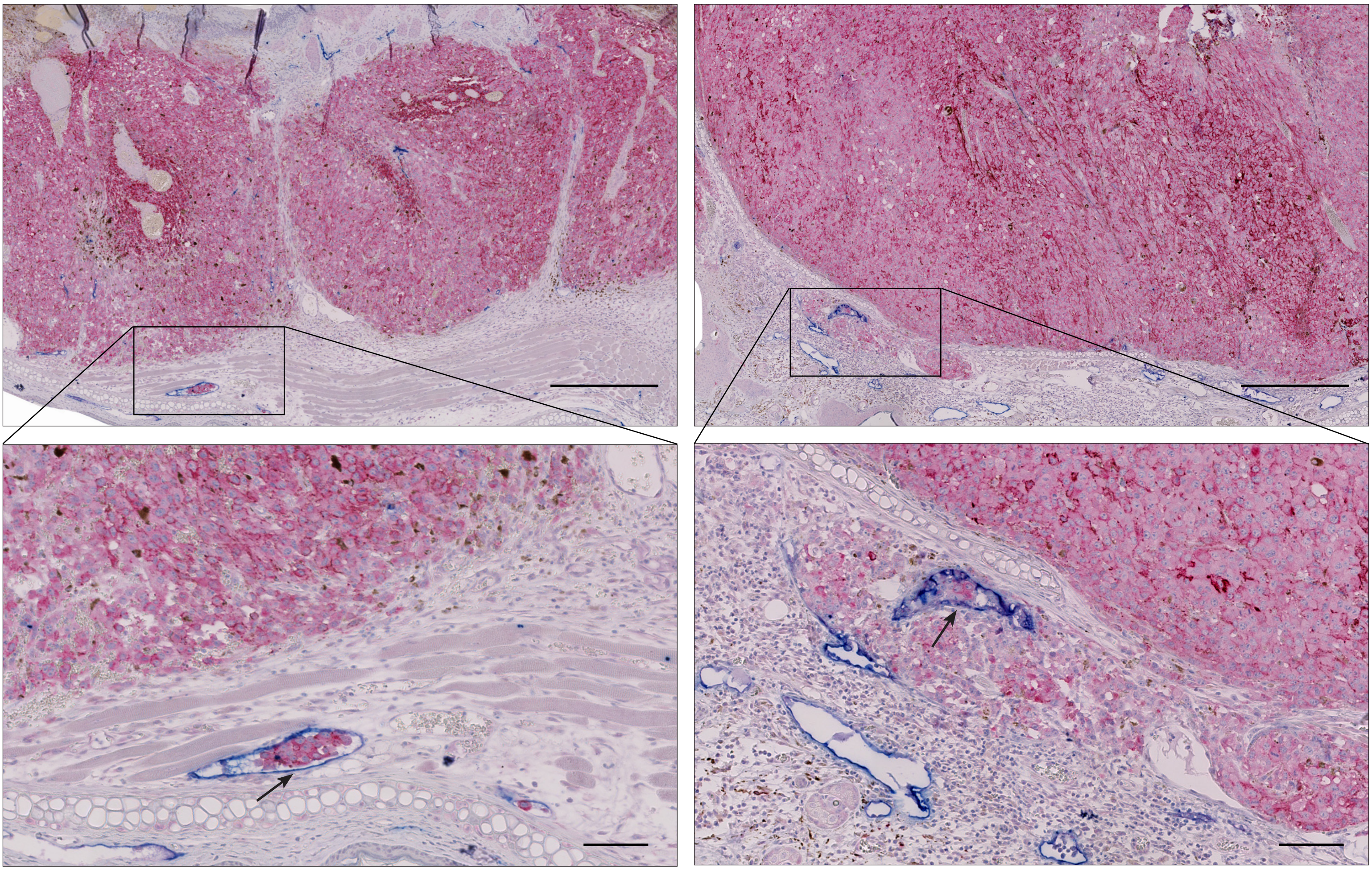

Supplemental Fig. SF3. Infiltrating tumor cells inside peritumoral lymphatic vessels in the primary tumor. Representative sponges populated with 1.5x10^5 B16F10Luc+ cells or 2x10^5 B16F10Luc+ were implanted in C57BL/6J mouse ears for 4 weeks. Lymphatic vessels (LYVE-1 positivity, blue staining) and tumor cells (tyrosinase activity, pink staining) were detected by immunohistochemistry in primary tumors. Lower panels represent a higher magnification of the region delineated by the square in the upper pictures. Black arrows indicate infiltrating tumor cells into lymphatic vessels. Scale bars represent 500 and 100 µm in the low and high magnification pictures, respectively.

Supplemental Fig. SF4

a

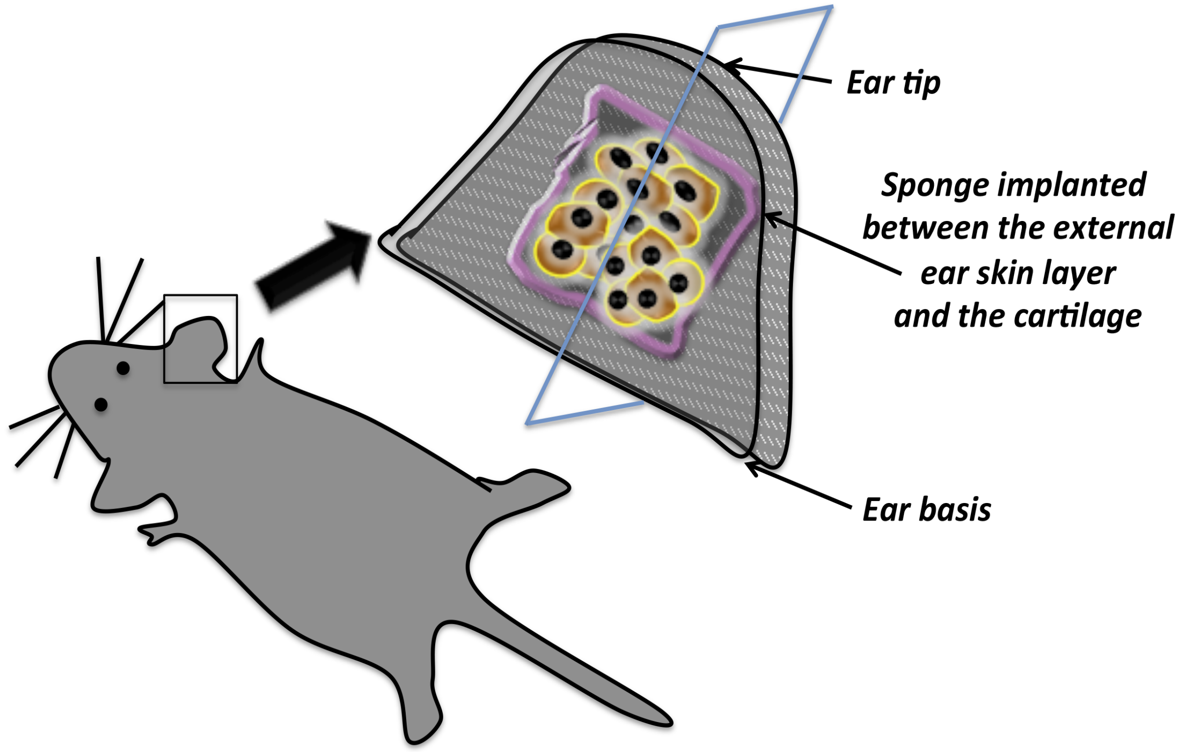

b

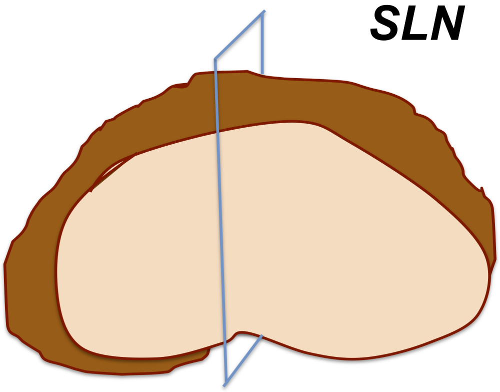

Supplemental Fig. SF4: Schemes of the mouse ear implanted with sponge and the mouse SLN. (a) Sponge was inserted in the central part of the mouse ear, between the external skin layer and the cartilage. (b) Drawing of the mouse SLN. After ear sponge and LN sectioning, the representative histological sections were taken from the central region of the sponge or the LN. In blue is indicated the position of these representative histological sections.

Supplemental Movie SM1. Ear sponge preparation.

Main steps in the preparation of the ear sponges.

Supplemental Movie SM2. Ear sponge implantation.

Main steps in the implantation of the sponges into mouse ears.
